# Interaction between Plasma Metabolomics and Intestinal Microbiome in db/db Mouse, an Animal Model for Study of Type 2 Diabetes and Diabetic Kidney Disease

**DOI:** 10.3390/metabo12090775

**Published:** 2022-08-23

**Authors:** Chenhua Wu, Jingjing Fei, Qing Xu, Yingjun Tao, Ziqi Zhou, Yurong Wang, Jie Wu, Harvest F. Gu

**Affiliations:** 1 Laboratory of Molecular Medicine, School of Basic Medicine and Clinical Pharmacy, China Pharmaceutical University, Nanjing 210009, China; 2 Laboratory of Minigene Pharmacy, School of Life Science and Technology, China Pharmaceutical University, Nanjing 211198, China

**Keywords:** diabetic kidney disease, intestinal–metabolic–kidney axe, metabolomics, microbiome, type 2 diabetes

## Abstract

Evidence has demonstrated that either metabolites or intestinal microbiota are involved in the pathogenesis of type 2 diabetes (T2D) and diabetic kidney disease (DKD). To explore the interaction between plasma metabolomics and intestinal microbiome in the progress of T2D-DKD, in the current study, we analyzed metabolomics in the plasma of db/db mice with liquid chromatography–mass spectrometry and also examined intestinal prokaryotes and entire gut microbiome dysbiosis at the genus level with both 16S rDNA and metagenomic sequencing techniques. We found that *Negativibacillus* and *Rikenella* were upregulated, while *Akkermansia*, *Candidatus*, *Erysipelatoclostridium* and *Ileibacterium* were downregulated in the colon of db/db mice compared with non-diabetic controls. In parallel, a total of 91 metabolites were upregulated, while 23 were downregulated in the plasma of db/db mice. The top five upregulated metabolites included D-arabinose 5-phosphate, estrone 3-sulfate, L-theanine, 3′-aenylic acid and adenosine 5′-monophosphate, and the five most significantly downregulated metabolites were aurohyocholic acid sodium salt, calcium phosphorylcholine chloride, tauro-alpha-muricholic acid sodium salt, galactinol and phosphocholine. These plasma metabolites were interacted with intestinal microbiomes, which are mainly involved in the pathways related to the biosynthesis of unsaturated fatty acids, fatty acid elongation, steroid biosynthesis, and D-arginine and D-ornithine metabolism. In the differential metabolites, N-acetyl-L-ornithine, ornithine and L-kyn could be metabolized by the correspondingly differential ontology genes in the intestinal metagenome. The current study thereby provides evidence for a gut–metabolism–kidney axis in the metabolism of db/db mice, in which the gut microbiome and circulating metabolomics interact, and suggests that information from this axis may contribute to our understanding of T2D and DKD pathogenesis.

## 1. Introduction

The International Diabetes Federation (IDF) Diabetes Atlas 9th edition provided the latest figures on diabetes worldwide. In 2019, approximately 463 million adults (20–79 years) were living with diabetes. The prevalence of diabetes is increasing in most countries. By 2045, the number of people with diabetes is estimated to reach 700 million [1]. Type 2 diabetes (T2D) is the most common form and accounts for about 90% of all diabetes cases. T2D is a complex disease in which genetic, environmental and metabolic risk factors are interrelated and contribute to its pathogenesis [1]. Accumulating evidence has demonstrated that gut microbiome dysbiosis is an additional risk factor in the rapid progression of insulin resistance in T2D [2,3]. Furthermore, thousands of metabolites derived from microbes interact with the epithelial, hepatic and cardiac cell receptors that modulate the host physiology, while changes in the gut microbiota can shift the host metabolism towards facilitated progression of T2D [4].

Diabetic kidney disease (DKD) is a main microvascular complication of diabetes and is characterized by albuminuria, a decline in glomerular filtration rate (GFR), hypertension, mesangial matrix expansion, glomerular basement membrane thickening and tubulointerstitial fibrosis [5,6]. Clinical observation has demonstrated that up to 40% of people with T2D will develop DKD. Furthermore, DKD has become the leading cause of end-stage kidney disease (ESKD), and patients need kidney replacement therapy to survive [6,7]. In current clinics of DKD, despite improvements in glycemic control and advances in reno-protective therapies, such as angiotensin-converting enzyme (ACE) inhibitors or angiotensin II receptor blockers (ARB), there is a large residual risk of ESKD onset and progression [8,9]. Therefore, widespread innovation is urgently needed to improve health outcomes for patients with T2D and DKD.

The db/db mice are characterized by hyperglycemia, obesity and urinary albumin excretion enhancement and have been widely used as a genetic rodent model for the study of T2D and DKD [10,11]. In the current study, we first evaluated db/db mice for diabetes and DKD through clinical indicators such as blood glucose levels, body weight, albuminuria as well as histopathological examination. We then performed metabolomics measurements of plasma samples collected from the mice and examined in parallel the intestinal prokaryotes and gut microbiome dysbiosis at the phylum and genus levels by using both 16S rDNA and metabolites sequencing protocols. Finally, we analyzed the interaction between plasma metabolomics and intestinal microbiome. Data from the current study may provide useful information for better understanding the intestinal–metabolic–kidney axis involved in the pathogenesis of T2D and DKD and, subsequently, for developing new biomarkers or targets for early diagnosis and therapeutical potential in these diseases.

## 2. Results

### 2.1. Basic Physio-Pathological Indicators

**Figure 1:**
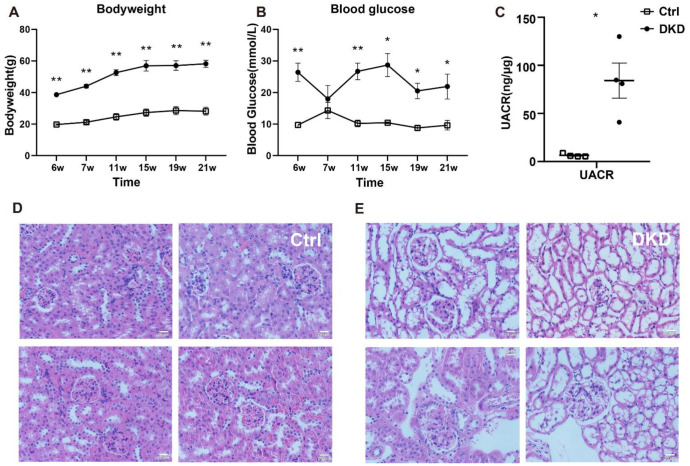
Clinical indicators, including body weight, blood glucose levels, histopathological examination and albuminuria in db/db mice. (**A**): Body weight (g) between the mice in the Ctrl and DKD groups from 6 to 21 weeks; (**B**): blood glucose levels (mmol/L); (**C**): UACR (ng/μg) in the mice of the Ctrl and DKD groups at the age of 21 weeks; (**D**,**E**): Images of HE stained kidney tissues from the mice in the Ctrl group and DKD group. Ctrl: control group; DKD: diabetic kidney disease; HE: hematoxylin–eosin; UACR: urinary albumin/creatinine ratio. *p* < 0.05 * and *p* < 0.01 **. Data are means with SE, n = 4. Scale bars = 20 μm.

Blood glucose levels, body weight and urinary albumin/creatinine ratio (UACR) values of the mice in Ctrl and DKD groups are represented in Figure 1A–C. The db/db mice in the DKD group had higher blood glucose levels, body weight and UACR values than the mice in the Ctrl group. Data demonstrated that db/db mice in the DKD group were not only obese and hyperglycemic but also had albuminuria [12]. Furthermore, HE staining analysis of kidney tissues indicated that damage of the glomerular structure, loss of renal cells, podocyte deficiency and other symptoms were seen in the db/db mice in the DKD group [13] (Figure 1E) compared with the mice in the Ctrl group (Figure 1D). The original data of blood glucose levels, body weight and UACR values are summarized in Supplementary Table S1.

### 2.2. Identification of Intestinal Prokaryotes

**Figure 2:**
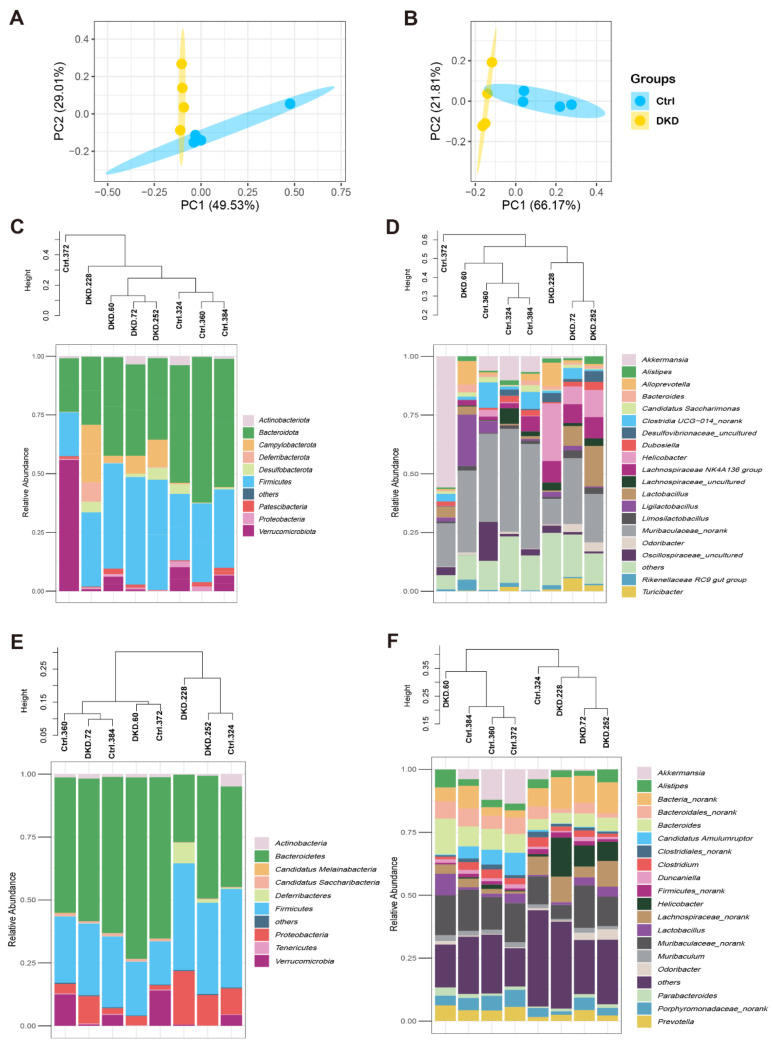
Composition of the intestinal microbiome in db/db mice detected with 16S rDNA and metagenomic sequencing analyses. (**A**,**B**) The relative abundances of 16S rDNA and metagenome at each genus level were used in Principal Component Analysis (PCA). X- and Y-axes represent the 1st and 2nd components of the PCA plot, respectively. (**C**,**D**): Community accumulation histogram showing the relevant abundance of top 10 phyla and top 20 genera detected by 16S rDNA sequencing. (**E**,**F**): Relevant abundance of top 10 phyla and top 20 genera detected by metagenomics.

After performing the bioinformatics analysis as described in the methods section, we obtained the relative abundance of each level of the intestinal microbiome with 16s rDNA (Supplementary Table S2A) and metagenomic sequencing (Supplementary Table S2B). To analyze the differences in intestinal microbiome between the Ctrl and DKD groups globally, a biplot of the generic-level PCA was created, as represented in Figure 2A,B. PC1 and PC2 showed approximately 80% variability (herein, data about prokaryotic content annotated by NR in the analysis of metagenomics are presented). Regarding the confidence intervals between the Ctrl and DKD groups, there was only a small overlap. We thus preliminarily concluded that the microbes of these two groups were different and further explored the data. The details of the composition and distribution of microbiome in the colon are shown in Figure 2C–F as community accumulation histograms of the relative abundance of the microbiome at the phylum and genus levels, as revealed by the analyses of either 16s rDNA sequencing or metagenomics, respectively. It was reported that *Bacteroidete* and *Firmicutes* comprise the majority (approximately 80%) of prokaryotic microorganisms, according to a previous study [14]. At the phylum levels, it was reported that T2D and obesity had a positive relationship with the relevant abundance of *Firmicutes* but negative with *Bacteroidete* [15]. Nevertheless, data from the current study suggest that there was no significant difference in the ratio of these two microbes between the Ctrl and DKD groups (Supplementary Figure S1A,B). At the genus level, *Muribaculaceae_norank*, *Akkermansia*, *Helicobacter* and *Lachnospiraceae* co-occurred in the top 10 genera, while *Muribaculaceae_norank* was the most numerous. In another finding, whether in phylum or genus levels, the microbiota in the Ctrl and DKD groups of mice showed specific distribution patterns in phylogenetic trees, which meant that the intestinal prokaryotes represented were homogeneous within the groups and heterogenous between the groups. However, the phylum levels, seen in clusters of metagenomes, between the Ctrl and DKD groups were chaotic; analysis of the phylum levels were obviously not suitable for inferring differences between these two groups.

### 2.3. The Genus Levels of Microbes in db/db Mice of DKD Group

**Figure 3:**
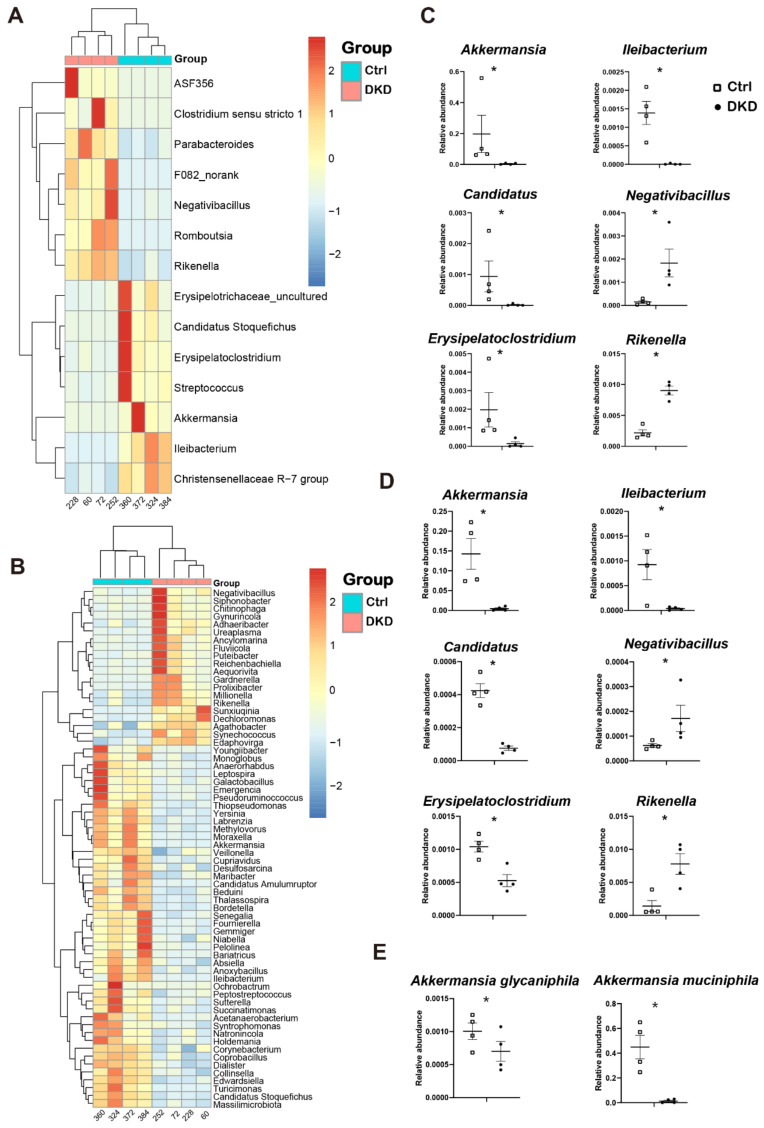
The gut microbiome in db/db mice underwent specific changes at the genus level, and the changes were repeatable between 16s rDNA and metagenomics. (**A**,**B**): Heat map of differential genera screened by Wilcox test according to 16S rDNA sequencing data (**A**) and metagenomics (**B**); (**C**,**D**): Point plots of genera with significant differences and consistent trends in 16S rDNA sequencing (**C**) and metagenomics (**D**); (**E**): Two species of *Akkermansia* and their expression between the Ctrl and DKD groups. * *p* < 0.05.

To reveal the key differences in microbes between the Ctrl and DKD groups, we carried out different analyses of either 16s rDNA sequencing or metagenomics at the genus level (Figure 3). Analysis results indicate that there was no significant change at the phylum level between the Ctrl and DKD groups, whereas there were dozens of significant variations at the genus level (*p* < 0.05). Due to the systematic difference of sequencing depth or annotation database between 16s rDNA and metagenomic sequencing technologies [16], the data from 16s rDNA sequencing implicated 14 genera with significant difference (Figure 3A), while metagenomic sequencing resulted in 68 genera (Figure 3B). Despite this, the technical duplication of six genera between these two different analyses was still found. In db/db mice of the DKD group, *Negativibacillus* and *Rikenella* were found to be upregulated, while *Akkermansia*, *Candidatus*, *Erysipelatoclostridium* and *Ileibacterium* were downregulated (Figure 3C,D), suggesting that these microorganisms may have potential associations with T2D and DKD. For instance, the abundance of *Akkermansia* measured in 16s rDNA and metagenomics sequencing was 48.26 and 32.41 times higher in the Ctrl group compared with the DKD group. The *Akkermansia* genus contains two species: *A. glycaniphila* and *A. muciniphila*. The results of metagenomics showed that the contents of these two species in the Ctrl group were 1.44 and 32.39 times higher than those in the DKD group (Figure 3E). There were significant differences in intestinal prokaryotes between the Ctrl and DKD groups, while technical duplication was achieved between these two groups. However, the specific biological significance of the difference in abundance of these species still needs to be further evaluated in combination with information about gene functional annotation from the metagenomic sequence and quantification of the metabolomics.

### 2.4. Intergroup Differences in Functional Annotations

**Figure 4:**
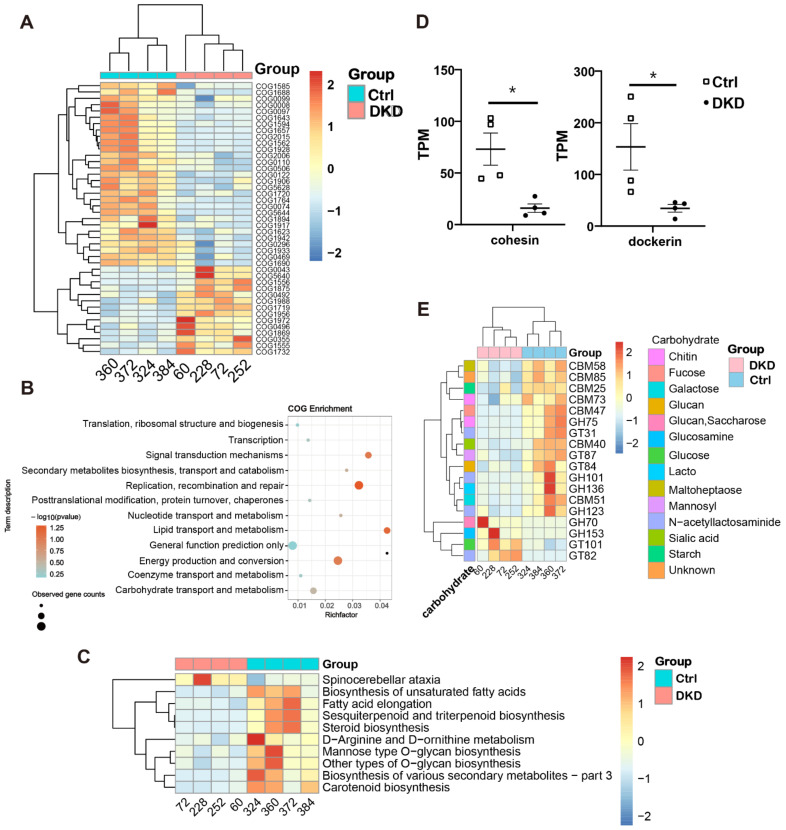
The annotation results from COG, KEGG and CARD databases showed that the metabolic capacity of DKD microbiome changed. (**A**): The heat map shows the COG annotation results with significance; (**B**): COG pathway enrichment analysis bubble diagram was obtained using the ClusterProfile package in R; (**C**): The heat map displays the level 3 sequence annotation results from the KEGG database; (**D**): In CAZy annotation results, transcripts per million (TPM) values of cohesin and dockerin are shown at the class level; (**E**): CAZy annotation results at the family level show TPM values in the heat map (scaled by rows). * *p* < 0.05.

Considering the limited information available from the changes in the relative abundance of microorganisms, the annotation of functional changes in the sequences of intestinal microbiome is necessary. We thus compared metagenomic sequences in COG [17], KEGG, CAZy [18] and CARD [19] databases to obtain the sequence annotations. Significantly different sequences obtained by COG annotation are shown in Figure 4A, while all original annotation information is represented in Supplementary Table S3. Among them, the top five COG sequences with the highest downregulation multiple in the DKD group were COG5644 (uncharacterized, log2FC = −8.82), COG1562 (ERG9, log2FC = −5.20), COG1928 (PMT1, log2FC = −4.79), COG1688 (RAMP, also known as DTL, log2FC = −3.80) and COG1764 (OsmC, log2FC = −2.73). On the contrary, COG1988 (predicted hydrolases, log2FC = 1.62), COG1719 (predicted hydrocarbon binding protein, log2FC = 1.63), COG0043 (UbiD, log2FC = 1.66), COG1972 (NupC, log2FC = 1.70) and COG5640 (serine protease, log2FC = 3.18) had the most increased multiples.

To explain the function of COG annotation data as described above more specifically, we used ClusterProfile [20] to conduct a COG pathway enrichment analysis. The enrichment analysis of the bubble chart (Figure 4B) informed us that lipid transport, carbohydrate transport and metabolism pathways had the highest rich factor. The level 3 KEGG pathway annotation difference showed that many secondary metabolite-related pathways, such as fatty acids, terpene, steroid, amino acid and polysaccharide, are more active in the colon of Ctrl group mice (Figure 4C). Considering that saccharide, the most important substances in organisms, is also a major cause of microvascular disease, including DKD, we displayed the class (Figure 4D) and family (Figure 4E) levels of the corresponding different sequences from the CAZy database obtained through BLASTP. At the class level, only cohesin and dockerin had significant differences, and both were increased in the Ctrl group (Figure 4D). At the family level, it is noteworthy that sequences related to N-acetyllactosaminide transport (GT31 and GH101) and degradation (GH123) all showed an obvious downward trend in DKD (Figure 4E). Sequences of metabolism (CBM73 and GH75) related to chitin, a derivative of N-acetyllactosaminide, similarly changed between the two groups. Moreover, it was obvious that most of the significantly different genes related to carbohydrate metabolism were downregulated in the DKD group. There were only four upregulated families in DKD, of which GH70, GH153 and GT101 are all related to glucose metabolism, and their substrates are Glucan (or Saccharose), Glucosamine and Glucose, respectively. 

### 2.5. Interaction between Plasma Metabolomics and Intestinal Microbiome

**Figure 5:**
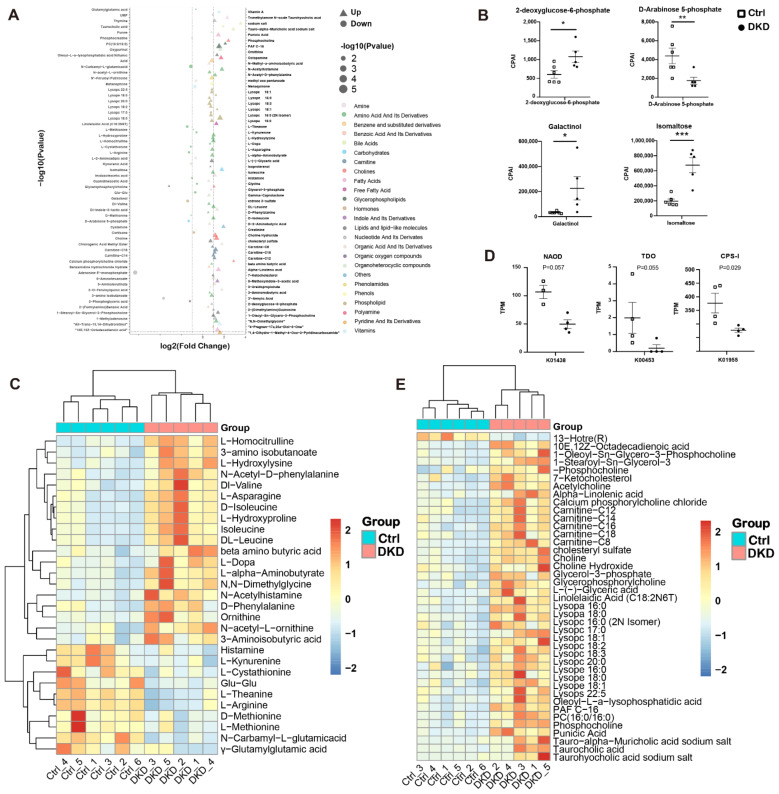
Changes in amino acids and lipid metabolites in plasma of db/db mice may be caused by intestinal microbiome. (**A**): Dot plots show the difference in multiples (horizontal axis) and significance (the larger the dot, the higher the significance) of all different metabolites (metabolites with *p* < 0.05 were retained) between the Ctrl and DKD groups. At the same time, different types of metabolites are distinguished by different colors. (**B**): Chromatographic peak area integral (CPAI) of four carbohydrate metabolites in the DKD and Ctrl groups (* *p* < 0.05, ** *p* < 0.01, *** *p* < 0.001). (**C**): A heat map shows CPAI of amino acid metabolites (metabolites with *p* < 0.05 and |log2FoldChange| > 0.05 were retained) in the Ctrl and DKD groups and scaled by rows. (**D**): The intergroup TPM value of the enzyme coding sequence annotated by KEGG. (**E**): A heat map of lipid metabolites, which is presented similar to Figure 5C.

In the current study, we attempted to find evidence of microbial influence on plasma metabolite content through functional annotation of the metagenome. In level 3 KEGG pathway annotation, we found that the metagenomic sequence changed significantly in the pathways related to the biosynthesis of unsaturated fatty acids, fatty acid elongation, steroid biosynthesis, D-arginine and D-ornithine metabolism, etc., between the two groups (Figure 4C). We conducted metabolomic analyses of plasma samples collected from the two groups of mice and found that the metabolism of db/db mice was closely related to the process of T2D and DKD. Metabolites discriminating the Ctrl and DKD groups (*p* < 0.05 and |LogFoldChange| > 0.5) are shown in Figure 5A. A total of 91 metabolites were found to be upregulated in DKD, while 23 were downregulated. The top five upregulated metabolites with maximal fold changes were D-arabinose 5-phosphate, estrone 3-sulfate, L-theanine, 3′-aenylic acid and adenosine 5′-monophosphate, while the five most significantly downregulated metabolites included aurohyocholic acid sodium salt, calcium phosphorylcholine chloride, tauro-alpha-muricholic acid sodium salt, galactinol and phosphocholine. As a pivotal indicator of kidney diseases, creatinine levels in plasma samples from the DKD group were 1.55 times higher than those from the Ctrl group. We thus summarized the effects and mechanisms of differentiated metabolites supported by evidence from the literature on DKD in Table 1. In this table, nineteen amino acid derivatives, six lipid derivatives and two carbohydrate derivatives in the differential metabolites were authentically associated with DKD, suggesting that they may play a role of alleviation or deterioration in the pathogenesis of DKD.

In carbohydrate metabolism, we found that galactinol, isomaltose, 2-deoxyglucose-6-phosphate and D-arabinose 5-phosphate were upregulated (Figure 5B). In amino acid metabolism, the plasma content of valine, leucine and isoleucine belonging to BCAA was higher in the DKD group compared with that in the Ctrl group (Figure 5C). We also found differences in the D-arginine and D-ornithine metabolic pathways in the KEGG annotation (Figure 4C). In the metabolome, downregulation of L-arginine and upregulation of N-acetyl-L-ornithine and Ornithine were displayed (Figure 5C), and the related encoding sequences of N2-acetylornithine deacetylase (NAOD) and carbamoyl phosphate synthetase I (CPS-I) decreased in the DKD group (Figure 5D). Furthermore, L-kyn was decreased in the DKD group (Figure 5C), while the gene abundance of tryptophan 2,3-dioxygenase (TDO) related to L-kyn generation was lower in the DKD group (Figure 5D). In lipid metabolism, a total of 42 different lipid metabolites were identified. Most of them were upregulated in DKD mice (Figure 5E), while only 13-hotre (R, 13-hydroxyoctadecatrienoic acid, a derivative of linoleic acid) was downregulated. Furthermore, we not only found that 2 lysophosphatidic acid (Lysopa) was elevated in the DKD group, but also detected that 6 lysophosphatidyl choline (Lysopc), 3 lysophosphatidyl ethanolamine (Lysope) and 1 lysophosphatidyl serine (Lysops) more broadly showed the same trend (Figure 5E).

**Figure 6:**
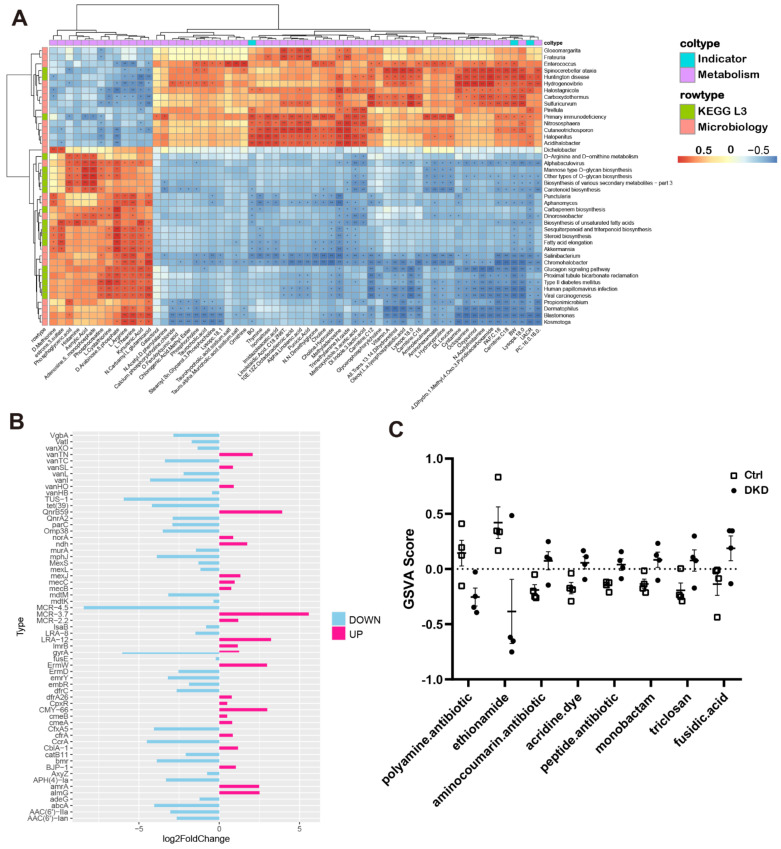
Correlations between DKD indicators, metabolites, relative abundance of microorganisms and microbial genes. (**A**): Correlation heat maps show the degree of association between clinical index test, metabolite CPAI, TPM values from KEGG L3 annotation in the metagenome and relative microbial abundance (* *p* < 0.05, ** *p* < 0.01). (**B**): The bar chart shows the different TPM multiples of bacterial (AMR) drug classes between groups. Up and down represent the changes in the DKD group compared with the Ctrl group. (**C**): The dot plots show the distribution of GSVA score annotated to AMR gene family sequences across the groups, and each antibiotic has a *p* value of less than 0.05.

We further calculated the Pearson coefficients among clinical indicators, metabolite CPAI, TPM values from the KEGG L3 annotation in the metagenome and relative abundance of microbiology to reflect the correlation between them (Figure 6A). The number of KEGG L3 Pathway and microbial genera significantly correlated with UACR was 15. Almost all metabolites had significant correlation with microorganisms, which provided a reference for the relationship between the intestine, metabolism and kidneys.

In CARD, there were a few million genomic resistance variants. Of them, 4498 with antibiotic resistance ontology (ARO) were supported by experimental publications, which can provide researchers with bacterial antimicrobial resistance (AMR) genes, antibiotic molecules, drug classes and related molecular mechanisms [19]. We thus analyzed the data based upon the comparison of the metagenomic sequences from the current study with the information from CARD, and the annotations for the AMR genes are represented in Figure 6B. We also performed a GSVA and obtained the GSVA score [21] of antibiotic resistance in the groups (Figure 6C) and the antibiotic-related AMR genes (Supplementary Table S4). The results of the analysis of differences, obtained using the generalized linear model, indicated that there were significant differences in the resistance of eight antibiotics between the Ctrl and DKD groups.

## 3. Discussion

In the current study, we provide evidence that the intestinal–metabolic–kidney axis exists in db/db mice, in which intestinal microbiomes and circulating metabolites are related and interact. We analyzed the intestinal prokaryotes and gut microbiome dysbiosis at the genus level using both 16S rDNA and metagenomic sequencing protocols (Figure 2). Data from both the PCA analysis and Bray–Curtis distance demonstrate that genus-level microbes are more competent to show the difference between DKD and Ctrl mice than phylum-level microbes. Six genera, including *Akkermansia*, *Ileibacterium*, *Candidatus*, *Negativibacillus*, *Erysipelatoclostridium* and *Rikenella*, had significant and repeatable inter-group differences. Of these six genera, *Akkermansia* and *Erysipelatoclostridium* have been reported to be associated with T2D or obesity. Meanwhile, we also identified the other four genera, which have not been reported. In the past decade, several research groups showed that there is a negative correlation between *A. muciniphila* abundance and overweight, obesity, T2D or hypertension [22]. Depommier et al. then conducted a randomized, double-blind, placebo-controlled pilot study on the administration of *A. muciniphila* in overweight/obese insulin-resistant volunteers. The outcomes of this clinical trial demonstrated that supplementation with *A. muciniphila* shows safety, tolerability and efficacy to improve metabolic parameters, such as insulin resistance, circulating lipids, visceral adiposity and body mass [22]. Kim et al. performed another clinical trial in T2D patients and found that metformin is partly attributable to the gut microbiome, and *Erysipelato clostridium* is negatively associated with metformin′s hypoglycemic effect in T2D patients [23], while the remaining four mechanisms involved remain to be explored. Thus, the current study has demonstrated that these six genera may be associated with DKD.

Many metabolites have relationships with diabetes and DKD. We thus summarized the evidence from the literature in Table 1 for further reference. For instance, many amino acids (e.g., L-Methionine, L-theanine, L-Cystathionine) act as antioxidants and maintain the balance of carbohydrate and lipid metabolism to protect the kidney but decrease in plasma of DKD [8,24]. In terms of lipid metabolites, all 40 lipids with significant differences, except 13-Hotre(R), were upregulated in the DKD group. Among them, the increase in L-Carnitine, 7-Kcho, Lysopa and their derivatives also indicates a more active inflammatory state, which may cause the activation of various immune cells, including Mac and T Cells [25,26]. However, many lipid derivatives (e.g., Carnitine, 7-KCHO, Isoproterenol) were elevated in DKD (Figure 5E), and it has been shown that some lipid derivatives have pro-inflammatory [26], pro-oxidative stress and pro-apoptotic effects [27]. However, carbohydrate derivatives other than glucose seem to play less important roles in DKD progression than amino acids and lipids (Figure 5B,C,E). For the significantly differentiated metabolites we found, we summarized and made Table 1 to reveal their association with DKD more intuitively.

Regarding the general adaptability and diversity of microorganisms, we believe that the sequence function of the microbiome is more significant than its abundance in DKD pathogenesis. We then performed functional annotation on metagenomic sequences using multiple databases to combine metabolomics to reveal the underlying mechanisms that influence DKD progression. The annotation by COG and KEGG indicated that the functions of metagenomic sequences in carbohydrate, amino acid and lipid metabolism fluctuated in DKD (Figure 4A–C,E).

**Figure 7:**
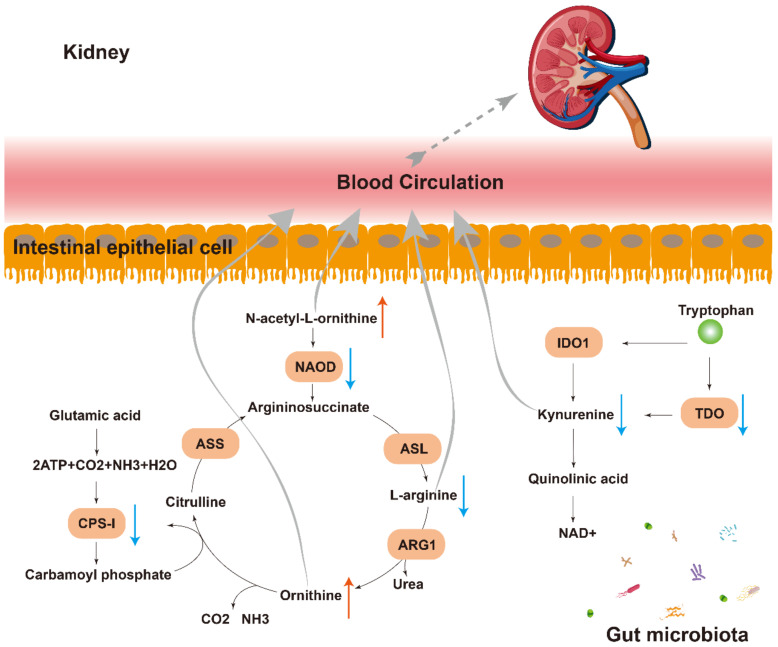
Pathways of communication between the microbiota and the kidney. Multiple indirect (e.g., N-acetyl-L-ornithine, kynurenine and L-arginine) pathways exist through which the gut microbiota can modulate the gut–kidney axis. NAOD: N2-acetylornithine deacetylase; IDO1: Indoleamine-2,3-Dioxygenase 1; TDO: Tryptophan 2,3-dioxygenase; ASL: Argininosuccinate lyase; ARG1: Arginase 1; CPS-I: Carbamoyl phosphate synthetase I; ASS: Argininosuccinate synthetase.

The result of the metabolome analysis showed that L-arginine was deficient when N-acetyl-L-ornithine and ornithine accumulated (Figure 5A,C). Coincidentally, N-acetyl-L-ornithine can be deacetylated by NAOD to form L-Arginine, which is the substrate for ornithine. In the current KEGG annotation, the NAOD encoding gene reads were found to be decreased in DKD (Figure 5D and Figure 7). In addition, encoding sequences of CPS-I decreased in the DKD group, while CPS-I can use ammonia, which is produced by ornithine, to synthesize carbamyl phosphate and participate in the ornithine cycle by converting it into citrulline. The decrease in CPS-I leads to a decrease in the synthesis of citrulline, which explains the upregulation of ornithine (Figure 5D and Figure 7). Moreover, as a tryptophan derivative, the L-Kyn level in the plasma of the DKD group was decreased, while the gene abundance of TDO, one of the rate-limiting enzymes of the Kynurenine pathway related to L-Kyn generation, was lower in the DKD group (Figure 5D and Figure 7). 

In this study, we found that the function of the metagenomic sequence has a certain effect on plasma metabolite content. For example, in terms of amino acid metabolism, there are more differential metabolites for DKD supported by the literature than carbohydrates and lipids (Table 1). Moreover, not only did the metabolic capacity of D-Arginine and D-ornithine decline in KEGG Level 3 annotation, but a decrease in the NAOD coding sequence was also found, which leads to an increase in Ornithine and a decrease in L-Arginine in the plasma of DKD; N-acetyl-L-Ornithine cannot be metabolized properly and thus accumulates in the plasma (Figure 5C,D and Figure 7). A balance of these two amino acids is also thought to be important for maintaining patients′ glucose tolerance levels [8]. A clinical observation has reported that N-acetyl-L-ornithine levels in subjects with diabetes and DKD were higher than those in individuals with normal glucose tolerance [28]. For another amino acid, the decrease in L-Kyn content in DKD may be related to the decrease in the TDO coding sequence (Figure 5D) and further correlated with proteinuria and inflammation [24].

To explore the biomarkers for the diagnosis and treatment of DKD, we analyzed the Pearson coefficients between UACR, blood glucose, microbial abundance and metabolites. The results showed that almost all metabolites were significantly correlated with microorganisms (Figure 6A), and there were a dozen genomic resistance variations in CARD (Figure 6B,C). In agreement, the most direct approach to microbial remodeling is antibiotic therapy. We thus focused on detection of AMRs and innovated the collation of antibiotic resistance targeted by various AMRs with GSVA analysis. The current study demonstrated that, among the changes in antibiotic resistance between groups, the polyamines with broad antibacterial spectrum may play a central role in the development of T2D-DKD. The antibiotics with increased resistance in the Ctrl group of this study may be expected to be used for assistant treatment in DKD. These microorganisms and antibiotics we found are expected to provide innovative ideas for the prevention and treatment of DKD as supplements.

In conclusion, we comprehensively monitored the progression of DKD, intestinal microbiome and plasma metabolic changes in non-diabetic control and db/db mice and provided evidence that the intestinal–metabolic–kidney axis exists in DKD. The results indicate that intestinal microbiome disturbances can directly or indirectly affect plasma metabolism and subsequently affect renal status and functions. Therefore, the data from the current study are useful for better understanding the pathogenesis of T2D and DKD.

## 4. Material and Methods

### 4.1. Animals and Physio-Pathological Parameters

Six-week-old male db/db (BKS.Cg-Dock7m +/+ Leprdb/J) mice and C57BL/6J mice were purchased from Cavens Laboratory Animal Co. Ltd. (Changzhou, China). The mice were kept in the barrier environment of the animal experiment center, Xuanwu campus, China Pharmaceutical University (CPU), and fed a normal pellet diet with water and food provided ad libitum for 4 weeks. Two groups of mice were retired breeders housed for at least 2 weeks before all experimental procedures. For the group of DKD mice, urine samples were collected by metabolic cage (DXL-XS, FENGSHI, Suzhou, China) for 24 h once a week. The urine samples were used to determine microalbuminuria (MAU) and creatinine (Cr); fresh urine was centrifuged (956× *g*, 10 min), and the supernatant was then fetched and stored at −80 °C. Concentrations of MUA and Cr were measured by ELISA quantitative kits (Elabscience Biotechnology, Houston, TX, USA) with Sandwich-ELISA and Competitive-ELISA principles, respectively. The db/db mice with DKD (DKD group) were diagnosed from a urinary albumin/creatinine ratio (UACR) greater than 30.0 ng/μg and were included in the DKD group, while the Ctrl group consisted of C57BL/6J mice with normal blood glucose levels and UACR values. 

After the mice were anesthetized with sodium pentobarbital (20 mg/mL, 50–70 mg/kg) at 21 weeks of age in the laboratory of molecular medicine, blood samples were collected from the mice by eyeball extraction, and kidneys were flushed with PBS via aortic catheterization at a speed of 6 ml/min until blanched. All left kidneys were placed in general purpose tissue fixative fluid (Servicebio, Wuhan, China) for hematoxylin–eosin (HE) staining. The colons of the mice were surgically ligated to ensure that they were free from contamination by environmental microorganisms and then stored in liquid nitrogen. All experiments with the mice were approved by the Institutional Animal Care and Use Committee of CPU. 

### 4.2. Metabolomic Analysis with Plasma Samples

For metabolomic analysis, 100 μL plasma was added with 4 times volume of pure methanol for the precipitation of protein. After vortex oscillation and ice incubation standing for 5 min, the supernatant was collected after high-speed centrifugation at 15,000× *g*, 4 °C for 5 min. The collection was centrifuged again in a centrifuge tube at 15,000× *g*, 4 °C for 20 min after adding 1/2 volume of mass spectrometry-grade water. Finally, the supernatant was collected for analyses of the targeted metabolomics using liquid chromatography–mass spectrometry (LC-MS) based on the highly sensitive SCIEX QTRAP^®^ 6500+ mass spectrometry platform (SCIEX, Framingham, MA, USA), and a blank control was prepared with 53% methanol solution containing 0.1% formic acid, while the pre-treatment process was the same as that of the experimental sample.

MS analyses were performed in the ExionLC™ AD system (SCIEX) combined with a QTRAP^®^ 6500+ mass spectrometer (SCIEX). Samples were injected onto a BEH C8 Column (100 × 2.1 mm, 1.9 μm) with a 30 min linear gradient at a flow rate of 0.35 mL/min for the positive polarity mode. As eluents, 0.1% Formic acid-water and 0.1% Formic acid-acetonitrile were used. The solvent gradient was set as follows: 5% B, 1 min; 5–100% B, 24.0 min; 100% B, 28.0 min; 100–5% B, 28.1 min; 5% B, 30 min. The QTRAP^®^ 6500+ mass spectrometer was operated in positive polarity mode with Curtain Gas of 35 psi, Collision Gas of Medium, Temperature of 500 °C, IonSpray Voltage of 5500 V, Ion Source Gas of 1: 55, Ion Source Gas of 2: 55. The parameters for negative polarity mode were the same as those for positive polarity mode except column (aHSS T3, 100 mm × 2.1 mm), linear gradient (25-min), flow rate (0.35 mL/min), solvent gradient (2% B, 1 min; 2–100% B, 18.0 min; 100% B, 22.0 min; 100–5% B, 22.1 min; 5% B, 25 min) and IonSpray Voltage (−4500 V).

### 4.3. Precondition of 16S rDNA and Metagenomics Sequencing

Colons were stored in liquid nitrogen for genomic DNA extraction. After a quick return to normal temperature in a 25 °C water bath, genomic DNA from the colon contents was extracted using the EZNA Stool DNA Kit (Omega Bio-tec, Norcross, GA, USA), and the extraction quality was observed by 1% agarose gel electrophoresis (AGE). One part of the extracted DNA was amplified by PCR using primers 341F (5′-CCTAYGGGRBGCASCAG-3′) and 806R (5′-GGACTACNNGGGTATCTAAT-3′) belonging to the V3–V4 variable region of 16S rDNA. Afterwards, the PCR products were examined by 2% AGE and recycled by gel-cutting using an AxyPrepDNA Gel Recovery Kit (Axygen, Union, CA, USA) for 16S rDNA sequencing (Illumina, San Diego, CA, USA). 

Another part of the fresh genome DNA samples was directly processed into 450 bp fragments by an ultrasonic crushing machine named Covaris M220 (Covaris, Woburn, MA, USA). Afterwards, the metagenomic library was prepared for PE450 high-throughput sequencing (Illumina, San Diego, CA, USA). Low-quality and mice genomic DNA reads were removed before analysis.

### 4.4. Bioinformatical and Statistical Analyses

To analyze the intestinal microbiome based upon data from the 16S rDNA sequencing, Trimmomatic-0.38 was used to carry out the quality control (QC) on the pair-end (PE), while FASTQ files were removed from the machine. Reads with tail mass less than 20 bases (window size was 10 nt) were filtered out during the process, and reads with length less than 50 bases were discarded after QC. OTU clustering of non-repeated reads after splicing under 97% similarity was implemented by Usearch (version 10). The Ribosomal Database Project (RDP) Classifier Bayesian algorithm was adopted to perform taxonomic annotation on Silva database for 97% similar level of OTU representative sequence alignment to obtain the corresponding species classification information (domain, phylum, class, order, family, genus, species) of each OTU.

For further analysis of the intestinal microbiome based upon the data from metagenome, the QC process was similar to the 16S rDNA section, except that the sequences with length less than 70 bp were deleted after mass pruning. Clean reads were assembled with MEGAHIT (version 1.2.9), and species taxonomic annotation was performed by Kraken2 (version 2.0.6-beta) to obtain abundance information. Then, nucleic acid sequences were clustered by CD-HIT (version 4.5.7) with 95% similarity and then translated into protein sequences by Transeq (version 6.6.0.0); after that, the annotated information was obtained from Clusters of Orthologous Groups of proteins (COG), Comprehensive Antibiotic Resistance Database (CARD), Non-Redundant Protein Sequence Database (NR) and Carbohydrate-Active enZYmes Databases (CAZy) using the BLASTP function of Diamond (version 0.8.36). Kyoto Encyclopedia of Genes and Genomes (KEGG) annotations at all levels were done through KofamScan based on KEGG Orthology and Hidden Markov Model. In the functional annotation, the pathway enrichment analysis was performed using ClusterProfile (Version 4.0.5), and bubble mapping was visualized with ggplot2 (Version 3.3.5) packages. GSVA was carried out through GSVA Packages (Version 1.34.0) in R, and GSVA score was obtained to conduct difference analysis through a generalized linear model in limma (Version 3.42.2).

SCIEX OS (version 1.4) was used to dispose the mass spectrum file, and the integration and correction of chromatographic peaks were performed. Urine metabolites data were identified by comparing the collision energy, parent ion, product ion, retention time and declustering potential of each substance recorded in the novogene database (novoDB), which was built by analytical standards. The relative quantities of metabolites represented by the area integration data of all chromatographic peaks were obtained while parameters were set as follows: minimum peak height, 500; gaussian smooth width, 1; signal/noise ratio, 5.

Considering that the relative abundance data of omics did not conform to normal distribution, the difference analysis mentioned in this paper was implemented by a rank sum test in R through the Wilcox test function. Subsequent visualization, such as Principal Component Analysis (PCA), was completed by ggord (version 1.1.6) in R (version 3.6.0) with relative abundance as input data.

## Figures and Tables

**Table 1:** Mechanisms of amino acids, lipids and carbohydrate metabolites in DKD.

Metabolites	Classification	Roles	Mechanisms	Regulation in Current Study
γ-Glutamyl glutamic	Amino acid derivative	Alleviation	It may enhance mitochondrial metabolism and insulin secretion.	Down
N-Carbamyl-L-glutamicacid	Amino acid derivative	Alleviation	Activates the urea cycle to prevent high blood ammonia.	Down
L-Methionine	Amino acid derivative	Alleviation/Deterioration	Moderate intake can maintain glucose and lipid metabolism homeostasis in T2D. However, excessive intake can lead to increased insulin resistance, oxidative stress and inflammation.	Down
D-Methionine	Amino acid derivative	Alleviation	Protects the kidneys through antioxidants.	Down
L-arginine	Amino acid derivative	Alleviation	Protects renal endothelial cells by participating in nitric oxide synthesis, subsequently increasing glomerular filtration rate and tubular reabsorption and reducing proteinuria.	Down
L-theanine	Amino acid derivative	Alleviation	L-theanine relieves liver and kidney damage by reducing oxidative stress, inflammatory response and apoptosis.	Down
Glu-Glu	Amino acid derivative	Alleviation	Similar to Glutamyl glutamic.	Down
L-Cystathionine	Amino acid derivative	Alleviation	L-cystathionine can inhibit mitochondria-dependent apoptosis.	Down
L-Kynurenine	Amino acid derivative	Alleviation	L-kynurenine can hinder oxidative stress and immune response.	Down
Histamine	Amino acid derivative	Alleviation/Deterioration	Although histamine is a well-known inflammatory mediator, there is also evidence that it may reduce kidney damage in glomerular basement membrane glomerulonephritis.	Down
3-Aminoisobutyric acid	Amino acid derivative	Alleviation	It has several effects, including improving inflammation, insulin resistance, glucose homeostasis and lipid metabolism.	Up
N-acetyl-L-ornithine	Amino acid derivative	Deterioration	The content of N-acetyl-L-ornithine in T2D patients with DKD increased significantly, and it is an important progressive factor of DKD.	Up
Ornithine	Amino acid derivative	Indirect effects	Polyamines formed after ornithine decarboxylation led to renal hypertrophy.	Up
L-Homocitrulline	Amino acid derivative	Deterioration	The formation of L-homocitrulline produces ammonia, which triggers cytotoxicity of macrophages.	Up
L-Hydroxylysine	Amino acid derivative	Deterioration	Excessive amounts of L-hydroxylysine can cause collagen deposition, which leads to thickening of the glomerular basement membrane.	Up
DL-Valine	Amino acid derivative	Marker	It is a marker of DKD mouse plasma induced by STZ.	Up
DL-Leucine	Amino acid derivative	Marker	Like DL-valine, it can be identified as a marker of DKD.	Up
Isoleucine	Amino acid derivative	Marker	Similar to DL-Valine and DL-Leucine.	Up
L-Dopa	Amino acid derivative	Deterioration	L-dopa can normalize filtration fraction and correct pre- and post-glomerular resistance by means of preferential post-glomerular vasodilatation as a precursor of dopamine synthesis.	Up
Alpha-Linolenic acid	Lipid derivative	Marker	Linolenic acid intake was negatively correlated with DKD in Brazilian T2D patients.	Up
13-Hotre(R)	Lipid derivative	Alleviation	13-Hotre(R) improves inflammation and oxidative stress, and low long-term 13-Hotre(R) intake is associated with the development of chronic kidney disease in T2D.	Down
Carnitine and its derivative	Lipid derivative	Marker and Alleviation	Carnitine, a derivative of methionine, increases circulatory levels in chronic renal failure, but inhibits tubular oxidative stress, interstitial fibrosis and apoptosis.	Up
Lysophosphatidylcholine and derivatives	Lipid derivative	Marker and Deterioration	As a pro-inflammatory signal, these substances can recruit macrophages to attack. The accumulation level and activation degree of receptors are positively correlated with UACR and glomerular hypertrophy.	Up
Isoproterenol	Lipid derivative	Alleviation/Deterioration	On the one hand, there is evidence that isoproterenol can promote vascular dilation and stimulate proximal tubule proliferation to protect kidney; on the other hand, there are studies supporting that isoproterenol can cause renal tubule injury by triggering oxidative stress and endoplasmic reticulum stress.	Up
7-KCHO	Lipid derivative	Deterioration	It is a kind of cholesterol derivative, which can induce vascular cell apoptosis by promoting oxygen and inflammation.	Up
2-deoxyglucose-6-phosphate	Carbohydrate derivative	Deterioration	It increases the activity of glucokinase causing hyperactivation of glucose metabolism.	Up
D−Arabinose 5−phosphate	Carbohydrate derivative	Marker	Circulation level of it increased in STZ modelled diabetic rats.	Up

## Data Availability

Data from the current study are contained within the Table in the article or in files in the Supplementary Materials. All researchers are allowed to re-mine the data with the permission of the corresponding author due to privacy.

## Supplementary Materials

The following supporting information can be downloaded at: https://www.mdpi.com/article/10.3390/metabo12090775/s1, Figure S1: At the phylum level, there was no significant difference in relative abundance of Bacteroidetes and Firmicutes between Ctrl and DKD groups; Figure S2: Evaluated that db/db mice through clinical indicators, detected metabolomics with plasma samples, examined the intestinal prokaryotes and gut microbiome dysbiosis by using both 16S rDNA and metagenomic sequenc-ing protocols; Table S1: Body weight, blood glucose levels, UACR in the mice of Ctrl and DKD groups; Table S2: Relative abundance of microorganisms in each sample at each classification level (domain, phylum, class, or-der, family, genus, species) obtained from 16S rDNA sequencing analysis (**A**) and metagenomes (**B**); Table S3: The annotated results of metagenomic sequences in COG, KEGG, CAZy and CARD databases; Table S4: This list can be used for GSVA analysis and display relationship between AMR and corresponding antibiotic resistance modified from recorded in the CARD; Table S5: This data contains compound characteristics, quantitative results, and information on differences between groups for each substance detected in metabonomics.

## Institutional Review Bord Statement

The study was conducted according to the guidelines of the Declaration of Helsinki and approved by China Pharmaceutical University (Approval Code: 2019-08-0003 and Approval Date: 2019-08-26).

## Abbreviations

7-KCHO	7-Ketocholesterol
AGE	agarose gel electrophoresis
AMR	antimicrobial resistance
ARO	antibiotic resistance ontology
CARD	comprehensive antibiotic resistance database
CAZy	carbohydrate-active enzymes databases
COG	clusters of orthologous groups of proteins
COXs	cyclooxygenases
CPAI	chromatographic peak area integration
Cr	creatinine
DKD	diabetic kidney disease
ECM	extracellular matrix
ESKD	end-stage kidney disease
G-3-P	glycerol-3-phosphate
GBM	glomerular basement membrane
HE	hematoxylin-eosin
HMC	human mesangial cells
IDF	International Diabetes Federation
LC-MS	liquid mass spectrometry
L-Kyn	L-Kynureniure
LOXs	lipoxygenases
LPAR	lysophosphatidic acid receptor
Lysopa	lysophosphatidic acid
Lysopc	lysophosphatidyl choline
Lysope	lysophosphatidyl ethanolamine
Lysops	lysophosphatidyl serine
MAU	microalbuminuria
MS	mass spectrometry
NAOD	N2-acetylornithine deacetylase
NMR	nuclear magnetic resonance spectroscopy
novoDB	novogene database
NR	non-redundant protein sequence database
PCA	principal component analysis
PE	pair-end
QC	quality control
RAAS	renin-angiotensin-aldosterone system
RDP	ribosomal database project
SCFA	short-chain fatty acids
TDO	tryptophan 2,3-dioxygenase
UACR	urinary albumin/creatinine ratio
