# Supplementary material for: Interaction between Plasma Metabolomics and Intestinal Microbiome in db/db Mouse, an Animal Model for Study of Type 2 Diabetes and Diabetic Kidney Disease
Source: Metabolites. 2022 Aug 23;12(9):775. doi: 10.3390/metabo12090775 (PMC9503368; doi:10.3390/metabo12090775)
Supplement: Supplementary file 1 [file metabolites-12-00775-s001.zip › metabolites-1840384-proofreading - Supplementary.pdf]

Supplemental information

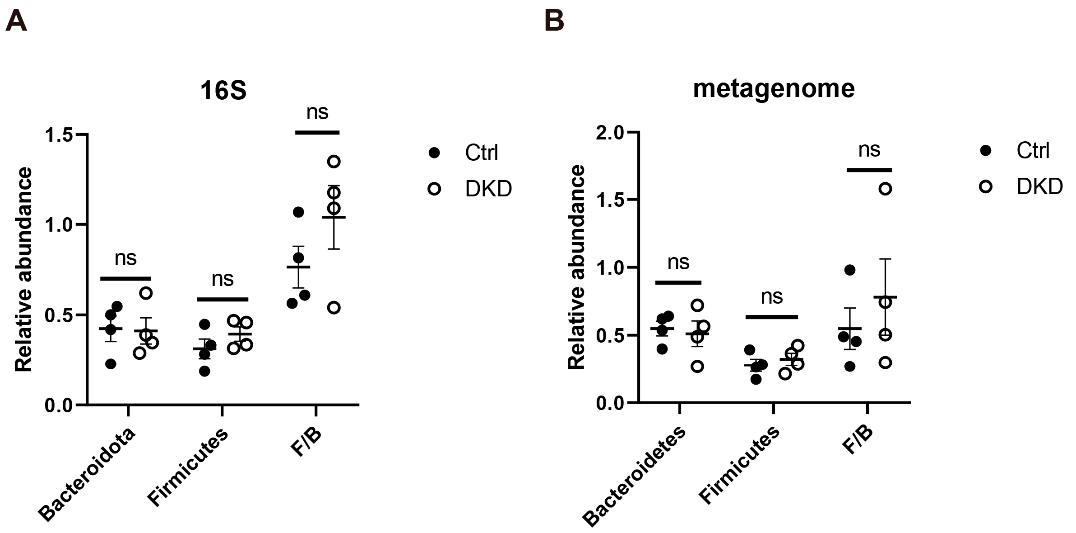

Supplemental Figure S1 At the phylum level, there was no significant difference in relative abundance of Bacteroidetes and Firmicutes between Ctrl and DKD groups

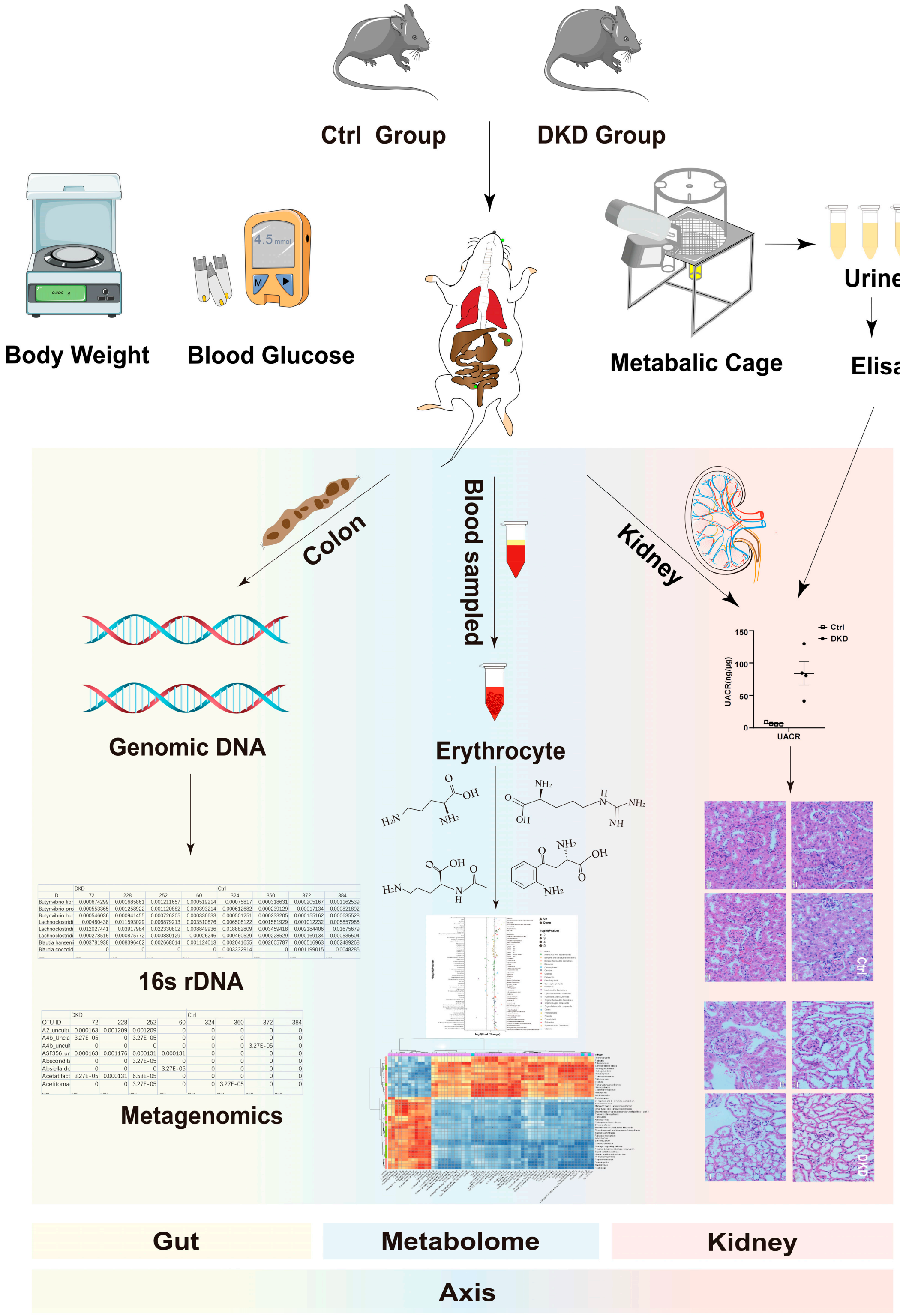

Supplemental Figure S2 Evaluated that db/db mice through clinical indicators, detected metabolomics with plasma samples, examined the intestinal prokaryotes and gut microbiome dysbiosis by using both 16S rDNA and metagenomic sequencing protocols

Supplemental Table S1

Body weight, blood glucose levels, UACR in the mice of Ctrl and DKD groups.

Supplemental Table S2A and B

Relative abundance of microorganisms in each sample at each classification level (domain, phylum, class, order, family, genus, species) obtained from 16S rDNA sequencing analysis (A) and metagenomes (B).

Supplemental Table S3

The annotated results of metagenomic sequences in COG, KEGG, CAZy and CARD databases.

Supplemental Table S4

This list can be used for GSVA analysis and display relationship between AMR and corresponding antibiotic resistance modified from recorded in the CARD.

Supplemental Table S5

This data contains compound characteristics, quantitative results, and information on differences between groups for each substance detected in metabonomics.
