# Supplementary figures and images for: Interaction between Plasma Metabolomics and Intestinal Microbiome in db/db Mouse, an Animal Model for Study of Type 2 Diabetes and Diabetic Kidney Disease
Source: Metabolites. 2022 Aug 23;12(9):775. doi: 10.3390/metabo12090775 (PMC9503368; doi:10.3390/metabo12090775)

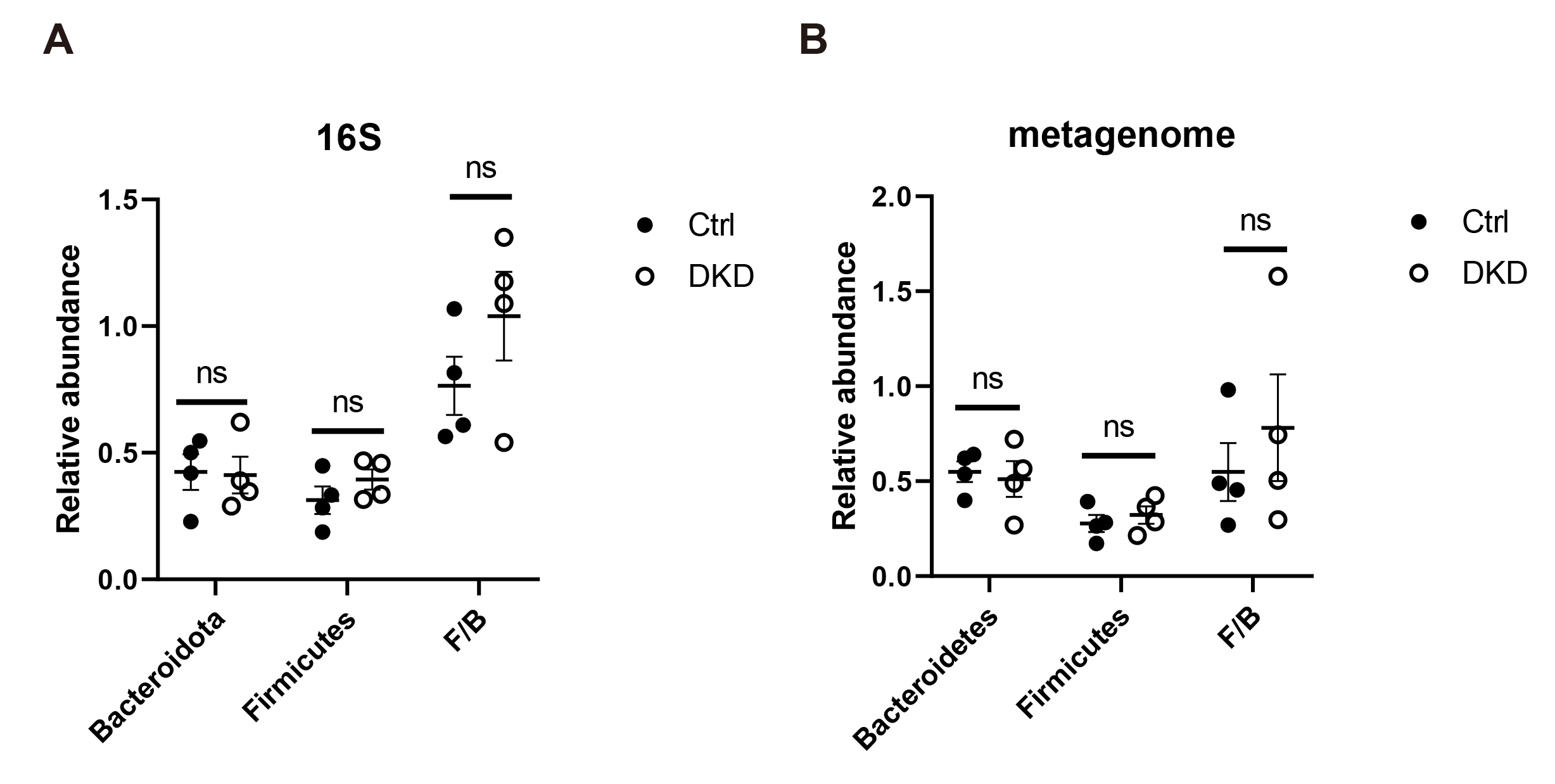

Supplement: Supplementary file 1 [file metabolites-12-00775-s001.zip › Supplemental Figure S1.tif]
